# Hypertriglyceridemia in female rats during pregnancy induces obesity in male offspring via altering hypothalamic leptin signaling

**DOI:** 10.18632/oncotarget.18519

**Published:** 2017-06-16

**Authors:** Tanzil Ur Rahman, Kamran Ullah, Zhang-Hong Ke, Meng-Xi Guo, Lu-Yang Jin, Jun Ren, Yu-Zhong Zhou, Yi Cheng, Xin-Yan Dong, Hai-Yan Pang, Ting-Ting Wang, Jian-Zhong Sheng, He-Feng Huang

**Affiliations:** ^1^ The Key Laboratory of Reproductive Genetics, Ministry of Education, Zhejiang University, Hangzhou, China; ^2^ Department of Pathology and Pathophysiology, School of Medicine, Zhejiang University, Hangzhou, China; ^3^ The International Peace Maternity and Child Health Hospital, School of Medicine, Shanghai Jiao Tong University, Shanghai, China; ^4^ Center of Reproductive Medicine, Fujian Maternity and Children Health Hospital, Fujian Medical University, Fuzhou, China

**Keywords:** triglyceridemia, leptin, hypothalamus, neuropeptide Y, pSTAT3

## Abstract

Maternal obesity influence the child's long-term development and health. Though, the mechanism concerned in this process is still uncertain. In the present study, we explored whether overfeeding of a high-fat diet during pregnancy in female rats altered metabolic phenotypes in an F1 generation and authenticated the contribution of hypothalamic leptin signaling. Leptin responsiveness and the number of immunopositive neurons for phosphorylated signal transducer and activator transcription 3 (pSTAT3) were analyzed. Neuropeptide Y in the arcuate nucleus of the hypothalamus and in nucleus tractus solitaries was examined. Triglycerides and leptin levels were increased in the high-fat diet mother. The number of neuropeptide Y positive cell bodies and neurons was significantly increased in the high-fat diet-F1 offspring (HDF-F1) as compared to Chow-F1. Leptin administration significantly decreased the food intake and increased the pSTAT3 expression levels in neurons in the arcuate nucleus of Chow-F1. However, leptin did not show any effect on food intake and had a reduced effect on pSTAT3 expression levels in neurons in the arcuate nucleus of HDF-F1. From the present domino effect, we conclude that mothers exposed to high-fat diet during pregnancy may pass the obese phenotype to the succeeding generation via altering hypothalamic leptin signaling.

## INTRODUCTION

Obesity is global health alarm and its frequency in adults and children is growing dramatically. Women with a high body mass index (BMI) are most likely susceptible to pregnancy complications and adverse perinatal outcomes including diabetes, hyperlipidemia, cardiovascular diseases, and numerous other problems [1, 2]. In the United States, one-third of women are obese and two-third are overweight [3]. In china, the proportion of overweight children and teenagers are 19.4% and 5.8 % are obese [4]. Research on different animal models revealed that HFD induced obesity and other metabolic disorders in their offspring [5, 6]. Moreover, maternal obesity is accountable for the direct transmission of obesogenic and diabetogenic phenotypes to the succeeding generation, but the exact mechanism is not fully understood.

The arcuate center (Arc) in the hypothalamus is a key area of leptin signaling [7, 8]. Peripheral leptin is transported transversely the blood-brain barrier (BBB) near to circumventricular organs to stimulate leptin receptor [9]. It has been reported that obesity and overnutrition modified the central leptin sensitivity [10] and appetite-controlling signals [6, 11]. These observations indicated that maternal obesity-programmed the brain development of their offspring, but the areas related to food intake and energy homeostasis has not been entirely explored.

Rodents increased their food ingestions, fat deposition, and insulin resistance during gestation [12, 13]. These features look like the metabolic adaptive conditions that cause obesity, for example, prolonged HFD feeding [14]. Remarkably, leptin sensitivity is a hallmark of both gestation and HFD feeding [15, 16]. Hence, leptin resistance could denote a crucial factor tangled in the metabolic alterations perceived during pregnancy.

Leptin, an adipocytes hormone, reduced food intake by binding to its receptor and then modifying the excitation of neurons in the Arc and nucleus tractus solitaries (NTS) in the brainstem [17, 18]. The Arc carries two major clusters of neurons that respond to leptin concentrations: pro-opiomelanocortin neurons (POMC) and neuropeptide Y (NPY) neurons. Anorexigenic peptides are articulated by POMC while orexigenic peptides are expressed by NPY [19, 20]. Leptin drops energy intake and body weight through pSTAT3 signaling pathway. Leptin phosphorylate and activate STAT3, afterward upregulates the gene encoding POMC and decreased NPY expression, and finally leads to a decrease in food consumption and body mass [21, 22]. Though most heavy humans and rodents have increased levels of circulating leptin, and despite leptin resistance, the high leptin levels have no effects on appetite and energy outflow [23].

The majority of research works were performed on males; it would be exciting to determine whether HFD feeding during pregnancy in female rats predispose obesity in their male offspring. We, therefore, asked a question, whether HFD feeding during pregnancy in female rats could alter feeding circuits and produce adiposity in male offspring and conclusive efforts may clarify the bottom line mechanism in the hypothalamus.

## RESULTS

### High maternal triglyceride (TG) levels are associated with persistent elevations in leptin levels

**Figure 1:**
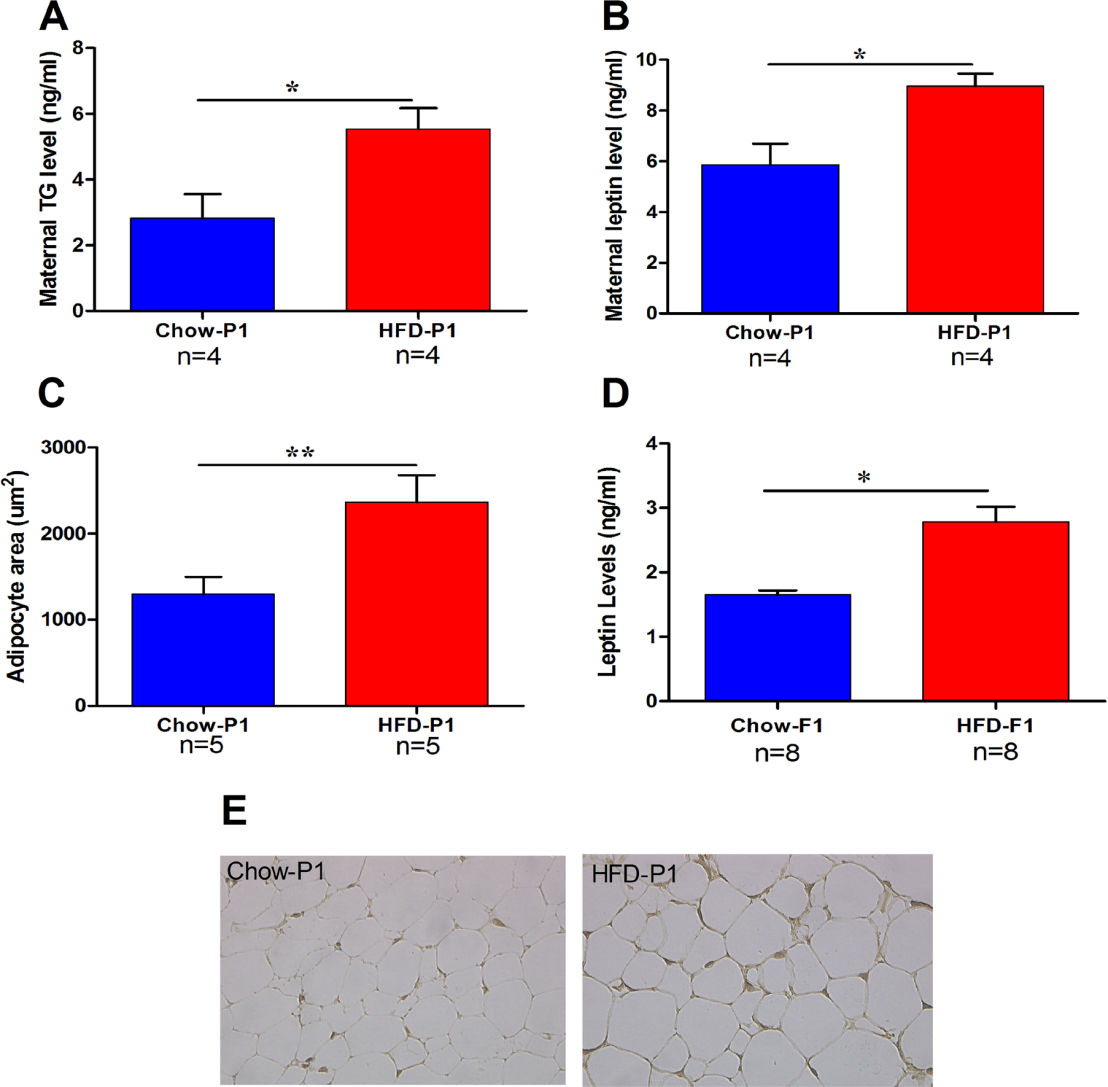
High maternal TG levels are linked with persistent elevations in male offspring leptin levels **(A)** TG levels in Chow and HFD mothers. **(B)** Serum leptin levels in Chow and HFD mother. **(C)** Adipocyte areas of the ovary fat pad of Chow and HFD mother. **(D)** Serum leptin levels of Chow-F1 and HFD-F1 offspring. **(E)** Representative photomicrographs of Chow and HFD mothers adipocytes. Results expressed as means ± SEM and the differences between the two groups were analyzed with Student's t-test. *p<0.05, **p<0.01.

To explore the effects of mTG on leptin levels in male offspring, we first investigated serum TG levels and leptin levels in both control mothers (Chow-P1) and high-fat diet mothers (HFD-P1). Serum TG and leptin levels in HFD-P1 rats were significantly increased than in controls (Figure 1A, 1B). On the other hand, the area of adipocytes was also significantly increased in HFD-P1 (Figure 1C). Moreover, we found that serum leptin concentration was also significantly increased in HFD-F1 offspring (Figure 1D) than that in Chow-F1 offspring. Figure 1E represents adipocyte photomicrographs of control and HFD mothers. These results revealed that maternal hypertriglyceridemia was associated with significantly increased leptin levels in their offspring.

### HFD feeding during pregnancy affects body mass, hyperphagia, and fat distribution

**Figure 2:**
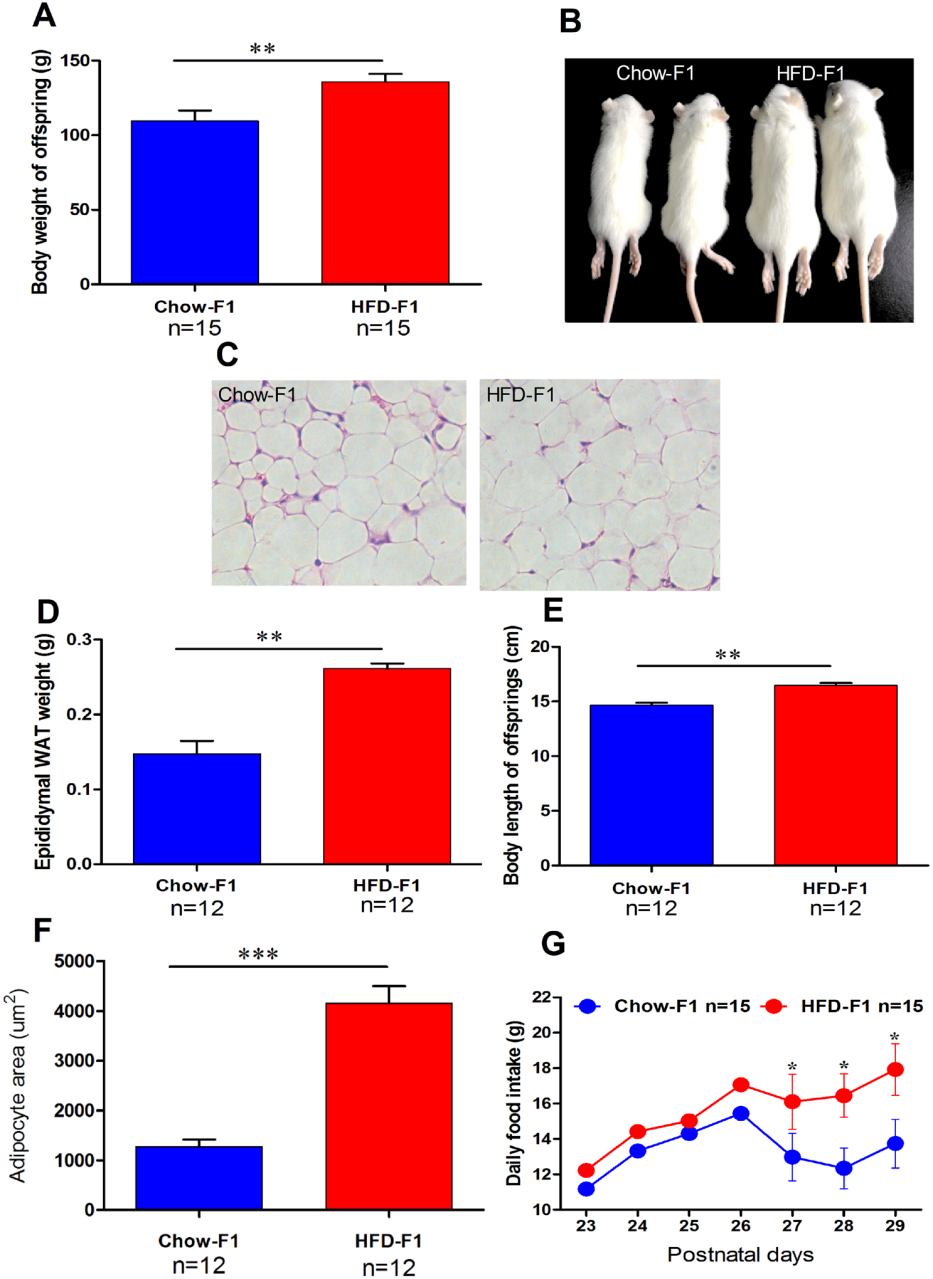
HFD feeding during pregnancy in female rats induced rapid weight gain, hyperphagia, and adiposity in male offspring **(A)** Body weight changes of Chow-F1 and HFD-F1 from postnatal day P0 to P28. **(B-C)** Representative photomicrographs of Chow-F1 and HFD-F1 offspring and adipocytes. **(D-E)** The Weight of epididymal WAT and body length of Chow-F1 and HFD F1. **(F-G)** Adipocytes area and daily food intake from P23 to P29. The data are expressed as the mean±SEM and the differences between the two groups were analyzed with Student's t-test. *p<0.05, **p<0.01, ***p<0.001.

To perceive whether maternal obesity-induced by HFD feeding during pregnancy in rat impact on the attaining body weight and developmental growth rate of their male offspring. The early body weight was the same between two groups. Later, HFD-F1 became heavier than the Chow-F1 up to day 29 (Figure 2A, 2B). Figure 2C represents adipocytes of Chow-F1 and HFD-F1. The weight of white adipose tissue on epididymis, body length, and adipocyte size were significantly increased in the HFD-F1 offspring (Figure 2D, 2E, 2F). At day 21 the male offspring were placed on solid chow diet and daily food intake was measured from day 21 to day 30. Daily food consumption by HFD-F1 was higher than Chow-F1 (Figure 2G). Blood glucose (Glu), total cholesterol (TC), low-density lipoproteins (LDL) and high-density lipoproteins (HDL) concentrations did not vary between HFD-F1 and Chow-F1 (Table 1). These data shows that maternal obesity as a result of HFD overfeeding during pregnancy induced significant adiposity possibly via an increased excitation of feeding circuits.

**Table 1:** Blood biochemical indices in male Chow-F1 and HFD-F1 offspring

		Glucose (mM)	HDL (mM)	LDL (mM)	TC (mM)
**Chow-F1**	n=8	7.8±0.37	0.80±0.06	0.78±0.01	2.35±0.09
**HFD-F1**	n=8	7.9±0.51	0.78±0.06	0.76±0.07	2.26±0.10

Data were presented as the mean±SEM. Glucose levels were measured using a glucometer. High-density lipoprotein, low density lipoprotein and total cholesterol were measured with a biochemical analyzer (Advia 2400 Chemistry System).

### HFD feeding during pregnancy displayed perverted neuronal feeding circuit

**Figure 3:**
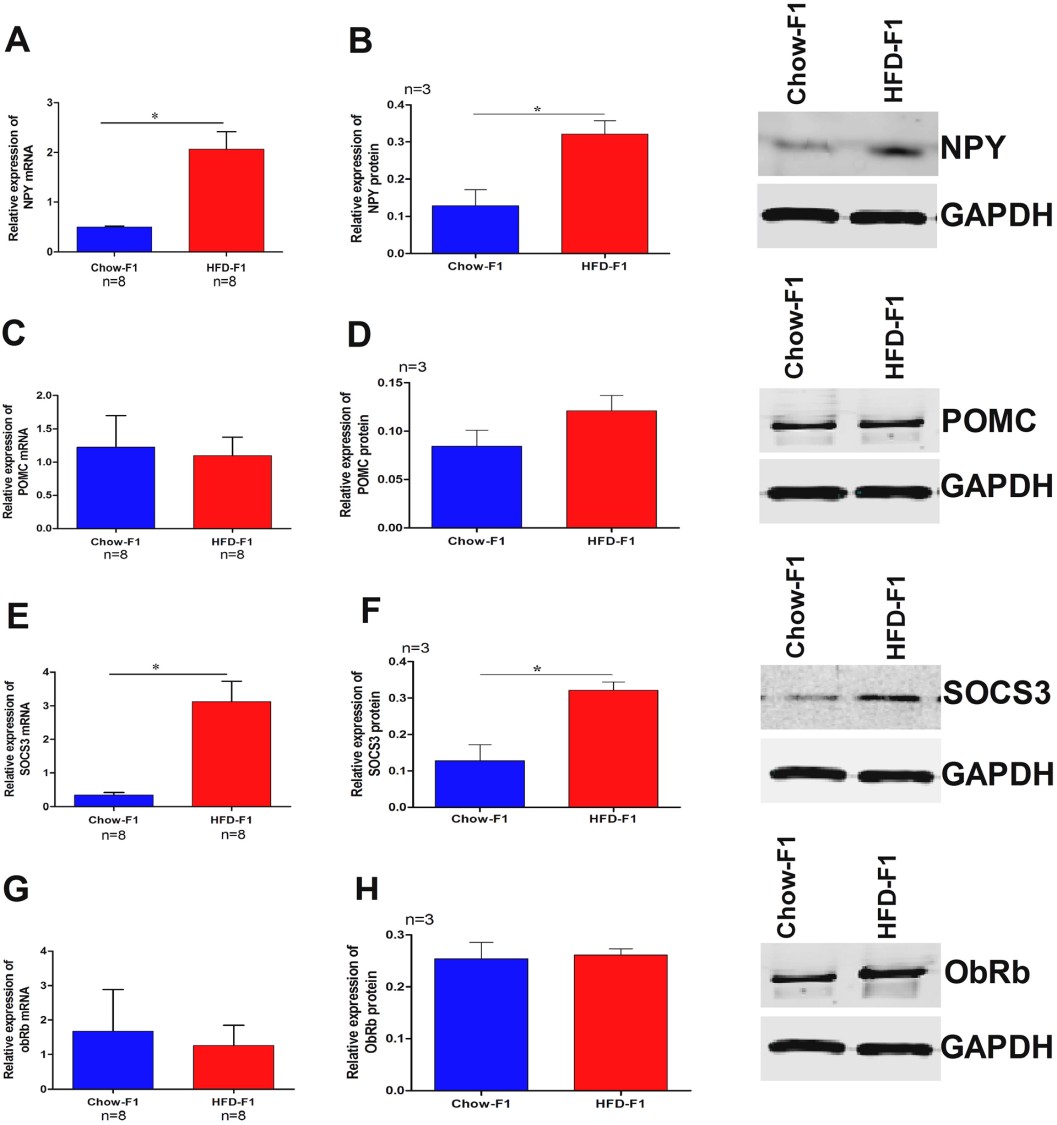
Effect of HFD overfeeding during pregnancy in female rats alters male hypothalamic feeding circuit genes The mRNA levels in the hypothalamus of Chow-F1 and HFD-F1 were determined by a quantitative real-time polymerase chain reaction and protein quantification by western blot. **(A-B)** Relative mRNA expression and proteins levels of NPY. **(C-D)** Relative mRNA expression and protein levels of POMC. **(E-F)** Relative mRNA expression and protein levels of SOCS3. **(G-H)** Relative mRNA expression and protein levels of ObRb. Values are expressed as the mean±SEM and analyzed with Student's t-test. *p<0.05.

Both POMC and NPY are hypothalamic peptides, and their expressions are increased and decreased by leptin signaling, respectively. Leptin signaling in the hypothalamus is negatively regulated by Suppressor of cytokine signaling 3 (SOCS3). To figure out if the hyperphagia of HFD-F1 was associated with these factors, we performed quantitative real-time PCR. NPY (Figure 3A) and SOCS3 mRNA levels (Figure 3E) of HFD-F1 (4 weeks old) were up-regulated, whereas POMC (Figure 3C) and the leptin receptor mRNA levels (ObRb) (Figure 3G) remains unchanged in both groups. Western blot analysis of hypothalamic protein extracts showed that HFD-F1 had increased NPY (Figure 3B) and SOCS3 (Figure 3F) levels than Chow-F1, while POMC (Figure 3D) and ObRb (Figure 3H) were not altered.

### HFD feeding during pregnancy displayed increased orexigenic NPY expression

**Figure 4:**
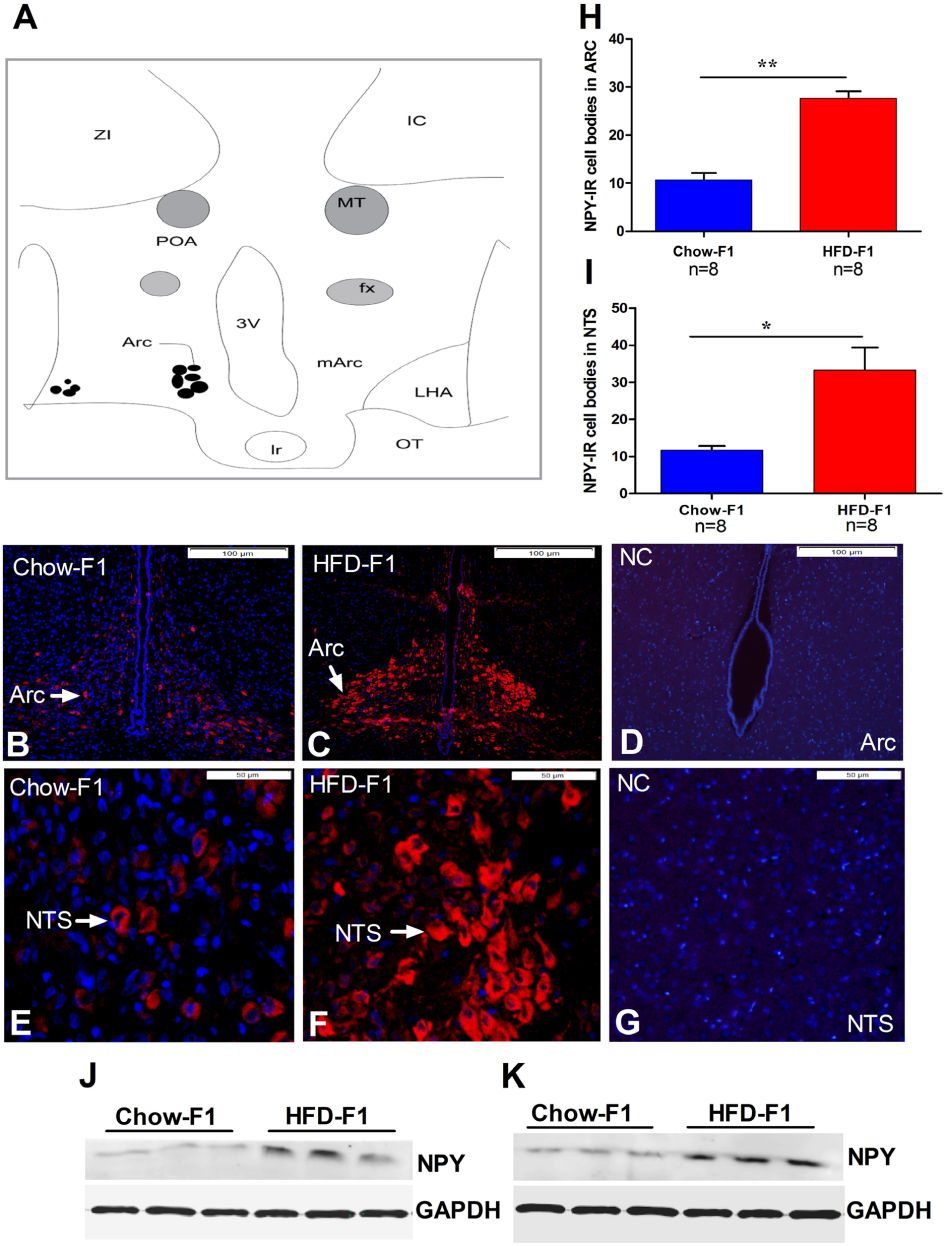
NPY expression in the hypothalamus and brain stem **(A)** Each solid circle represents NPY-IR cells. POA, preoptic area; OC, optic chiasm; fx, fornix; ZI, zona incerta; mARC, middle arcuate; LHA, lateral hypothalamic area; OT, optic tract; 3V, 3rd ventricle; IC, internal capsule; mt, mammillary tract. **(B-C)** NPY expression in the Arc of Chow-F1 and HFD-F1. **(E-F)** NPY expression in the NTS of Chow-F1 and HFD-F1. **(D-G)** Represents the negative control (NG) for Arc and NTS. **(H-I)** Quantitative results of NPY positive neurons in the Arc and NTS. **(J-K)** Western blot analysis of NPY protein from Arc and NTS. Loading was normalized using GAPDH. The data are expressed as the mean±SEM and the differences between the two groups were analyzed with Student's t-test. *p<0.05, **p<0.01.

To further evaluate the NPY-immunoreactivity (IR) in the Arc and NTS, immunofluorescence was performed. Figure 4A indicates the area of NPY-IR cell bodies in the Arc. Each solid circle represents NPY-IR cell bodies. Arc and NTS from HFD-F1 displayed a significantly increased number of NPY positive neurons (Figure 4C, 4F) than Chow-F1 (Figure 4B, 4E). Figure 4D, 4G signifies the negative control. Arrowhead represents the neuronal cell bodies in the Arc and NTS. Quantitative analysis showed that HFD-F1 had significantly increased the number of positive neurons in both Arc and NTS nuclei (Figure 4H, 4I). Immunohistochemical (IHC) analysis also showed the increased number of NPY-IR neuronal bodies in Arc and NTS of HFD-F1 offspring than Chow-F1 (Supplementary Figure 1). For more validation, we extracted protein from Arc and NTS and performed western blot analysis. Figure 4J, 4K shows that NPY protein concentrations in Chow-F1 Arc and NTS were significantly decreased as compared to HFD-F1.

### HFD feeding during pregnancy induced significant leptin resistance

**Figure 5:**
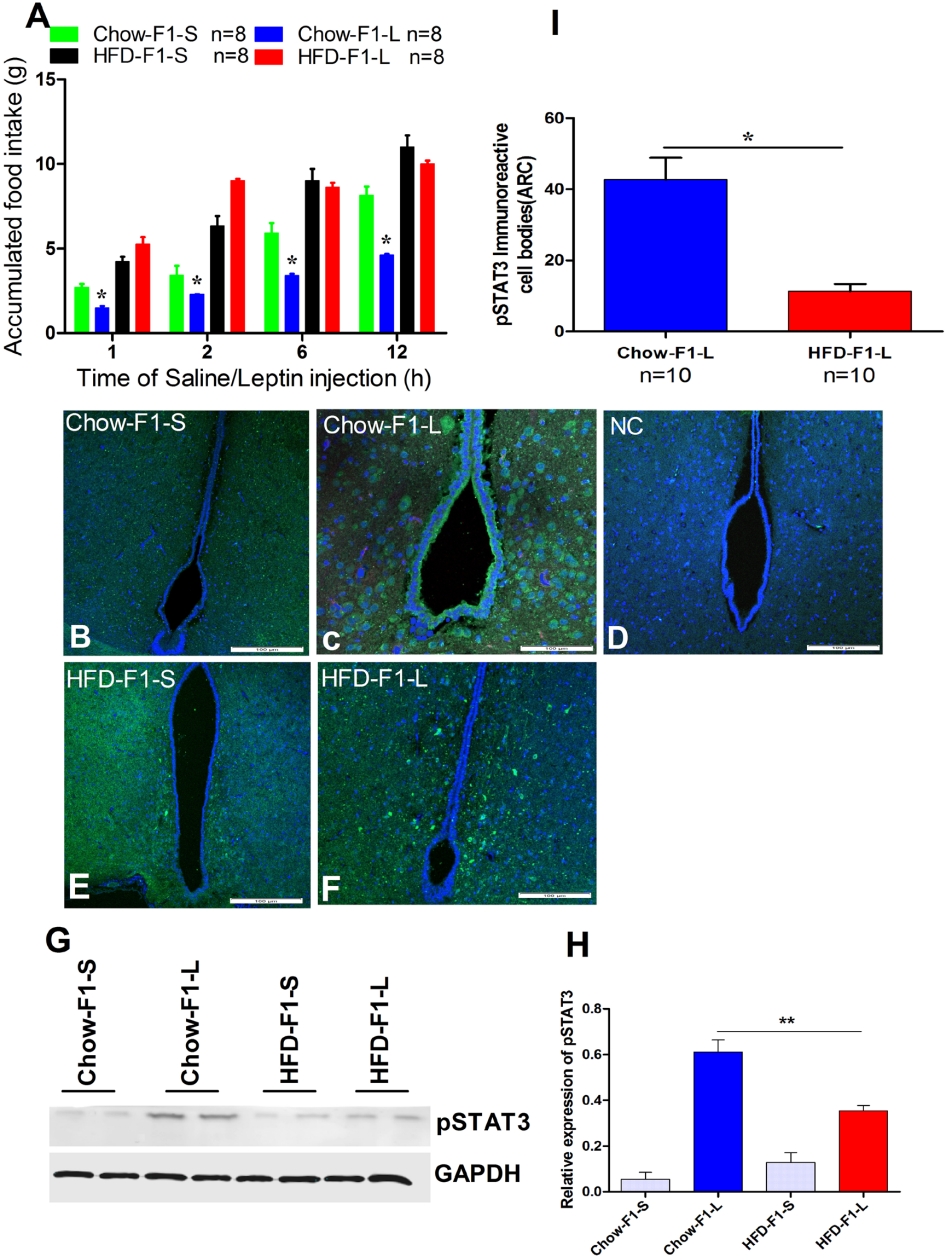
HFD overfeeding during pregnancy in female rats induced leptin resistance in male offspring Using a two-way ANOVA, food intake values at four-time points were found to be significantly lower in Chow-F1 treated with leptin compared to all other groups. *P < 0.05. **(A)** Food intake 1, 2, 6 and 12 h after i.p. leptin (2 μg/g)/saline. **(B)** Expression of pSTAT3 in Chow-F1 after saline injection. **(C)** Expression of pSTAT3 in Chow-F1 after leptin injection. **(D)** Represents the negative control (NC). **(E)** Expression of pSTAT3 in HFD-F1 after saline injection. **(F)** Expression of pSTAT3 in HFD-F1 after leptin injection. **(G-H)** Western blot analysis of pSTAT3 after leptin/saline injection. Loading was normalized using GAPDH and densitometry was performed using ImageJ software. One-way ANOVA was used to analyze the leptin and saline-treated groups, **p<0.01. **(I)** Quantitative results of pSTAT3 positive neurons after saline/leptin treatment. Data are expressed as the mean±SEM and the differences between the two groups were analyzed with Student's t-test. *p<0.05, **p<0.01.

To investigate if the hyperphagia of HFD-F1 is linked with leptin sensitivity in the hypothalamus, both food intake and quantification of pSTAT3-IR in Arc were achieved. In Chow-F1, leptin administration (2 μg/g) lowered food intake at 1, 2, 6 and 12 h subsequently. On another side, leptin injection has no significant effect on food intake in HFD-F1, suggesting that HFD-F1 might have leptin resistance (Figure 5A). On day 30, pSTAT3-IR neurons were very few or even invisible in the Chow-F1-S and HFD-F1-S groups (Figure 5B, 5E). After leptin injection, the Chow-F1 showed significantly increased pSTAT3 expression in the Arc (Figure 5C), whereas the HFD-F1 displayed a severe decrease of the Arc pSTAT3 activation (Figure 5F, 5I). Figure 5D represents the negative control. IHC results also displayed the decreased number of pSTAT3 IR cell bodies in the Arc of Chow-F1 offspring after leptin administration than HFD-F1 offspring (Supplementary Figure 2). Western blot analysis (**p<0.01) also confirmed that pSTAT3 protein level was significantly increased in the Arc of Chow-F1 offspring after leptin administration, and this effect was significantly reduced in HFD-F1 (Figure 5G, 5H). These results reveal that HFD over diet during pregnancy in female rats tempts substantial leptin resistance in their male progenies.

## DISCUSSION

The current results declared that HFD over intake during pregnancy in female rats could cause profligate weight gain and obesity in male offspring. This obese phenotype could be conceded into their second filial generation. Pups from HFD fed mother during pregnancy exhibited hyperphagia and increased body fat. Besides, offspring also showed significant alterations in hypothalamic leptin actions that might describe the deceptive leptin resistance.

Our results confirm that NPY and SOCS3 mRNA levels were higher in the HFD-F1, while POMC and ObRb mRNA levels were remains unchanged. Data from previous investigations disclosed that NPY level is higher in obese rats [24]. A study revealed that central NPY mRNA levels are high in some obesity models, like after temporary nutritional scarcity [25], lasting calories restriction [26], heavy exercise [27], and seasonal hyperphagia before hibernation [28]. These observations indicated that HFD feeding increased the NPY mRNA levels in the hypothalamus. SOCS3 has been suggested to cause defective leptin signaling in the Arc of HFD mice [29]. The heterozygous SOCS3-deficient mouse was more sensitive to the weight-reducing effects of leptin and was resistant to the development of diet-induced obesity [30]. Moreover, using an SOCS3 deficient mouse, Mori et al. showed that leptin-induced POMC level was increased in SOCS3 deficient mice than in wild-type mice [31]. These data designate that leptin-induced SOCS3 is a negative regulator of pSTAT3 signaling in the Arc. Data from previous research on mice by Pablo JE et al. presented that neither POMC mRNA expression nor ObRb expression in Arc was significantly different between HFD-induced obese mice and normal diet mice [32]. Our current results displayed that ObRb mRNA and POMC mRNA expression levels were not different between Chow-F1 and HFD-F1. These results agree with some studies in male mice fed with HFD [29, 33]. Sahu et al. indicated that ObRb expression in rats remained unchanged despite adenovirus-mediated hyperleptinaemia [33]. From all these observations, we conclude that excessive activity of SOCS3 is the potential mechanism for the leptin resistance in HFD-F1.

**Figure 6:**
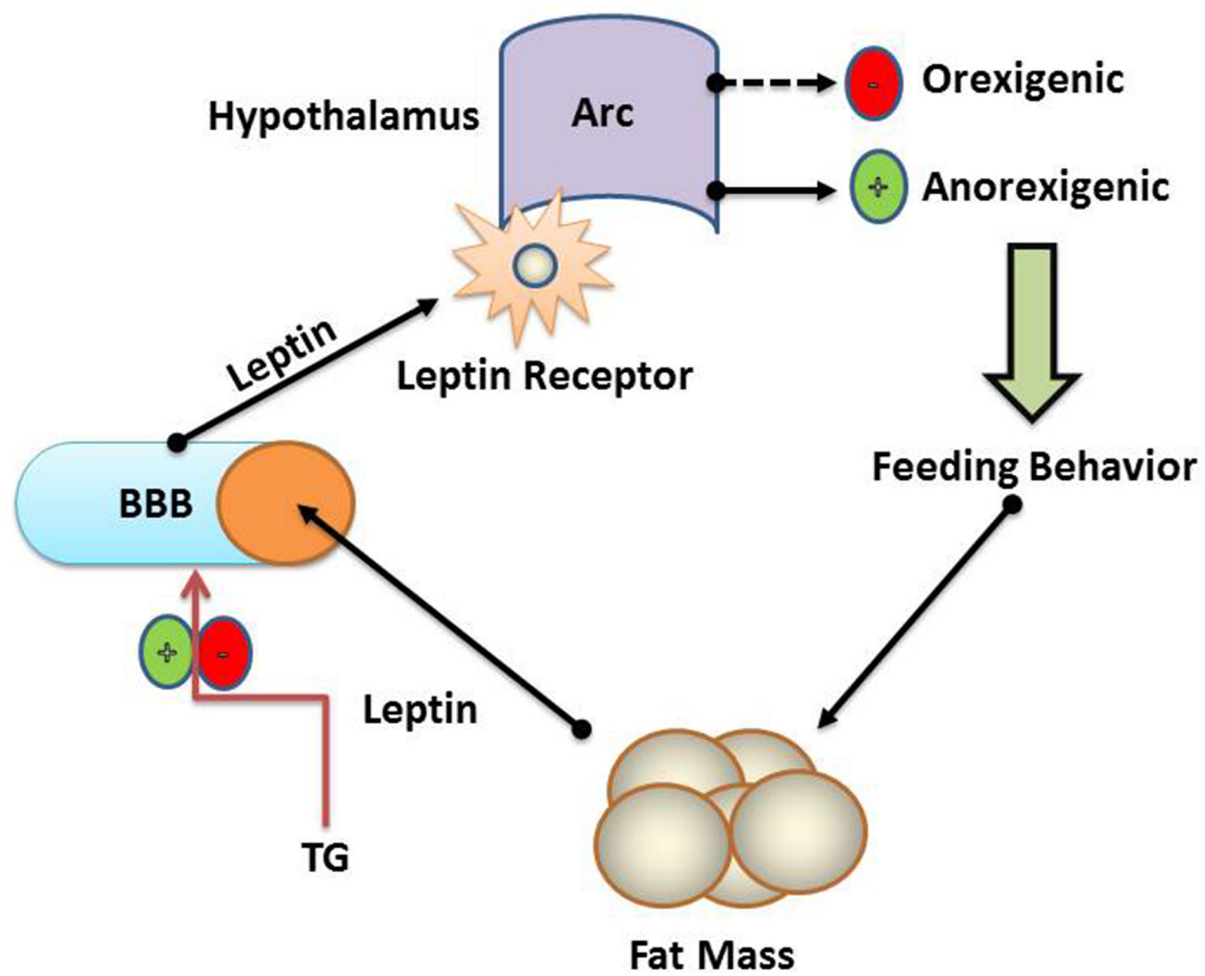
Schematic representation of TG and blood brain barrier Fat mass acts as a negative feedback loop for leptin. Increased fat mass results in increased levels of leptin in blood. TG controls the rate of passage of leptin across BBB. A saturable transport system at the BBB transports leptin into the hypothalamus' arcuate nucleus where leptin stimulates anorectics and inhibits orexigenic peptides, thus regulate feeding. TG and BBB have a critical role in the regulation of hypothalamic genes expression and control of feeding behaviors.

Our results revealed that high TG levels during pregnancy might induce chronically elevated leptin levels in offspring. It has been shown that TGs suppressed leptin passage through BBB and this blockade could be the reason for peripheral leptin sensitivity [34]. Adipose tissue release leptin into circulation and transported across BBB to the brain [35]. Their leptin binds to its receptor located in the Arc (Figure 6). Saturable transporter carries leptin across the BBB and defective transport may lead receptor errors and deteriorates with the increasing obesity [36]. The affinity of serum leptin levels and cerebrospinal fluid in obese humans [37] indicates that impaired BBB transport might account for overall leptin resistance than the receptor defects [9]. Data from clinical and animals studies showed that these metabolites were markedly elevated by both acute and chronic HFD situations [38]. Another study indicated that TG hydrolysis caused the release of fatty acids, which could modulate hypothalamic peptide expression [39]. It has been reported that hypothalamic injection of NPY increased TG levels [40]. These investigations showed that NPY neurons and cell bodies interact with peripheral metabolites via the hypothalamus.

Though HFD offspring from obese mother grew earlier than control pups and rapid weight gain after birth, observed in the present study, is because of metabolic remodeling of neuronal feeding circuits during pregnancy, which can further arouse food ingestion and increased body mass [41]. Because overfeeding plays a vital protagonist in obesity endemics [42]; therefore, we examined food intake in the F1 generation when the offspring were placed on standard chow diets.

**Figure 7:**
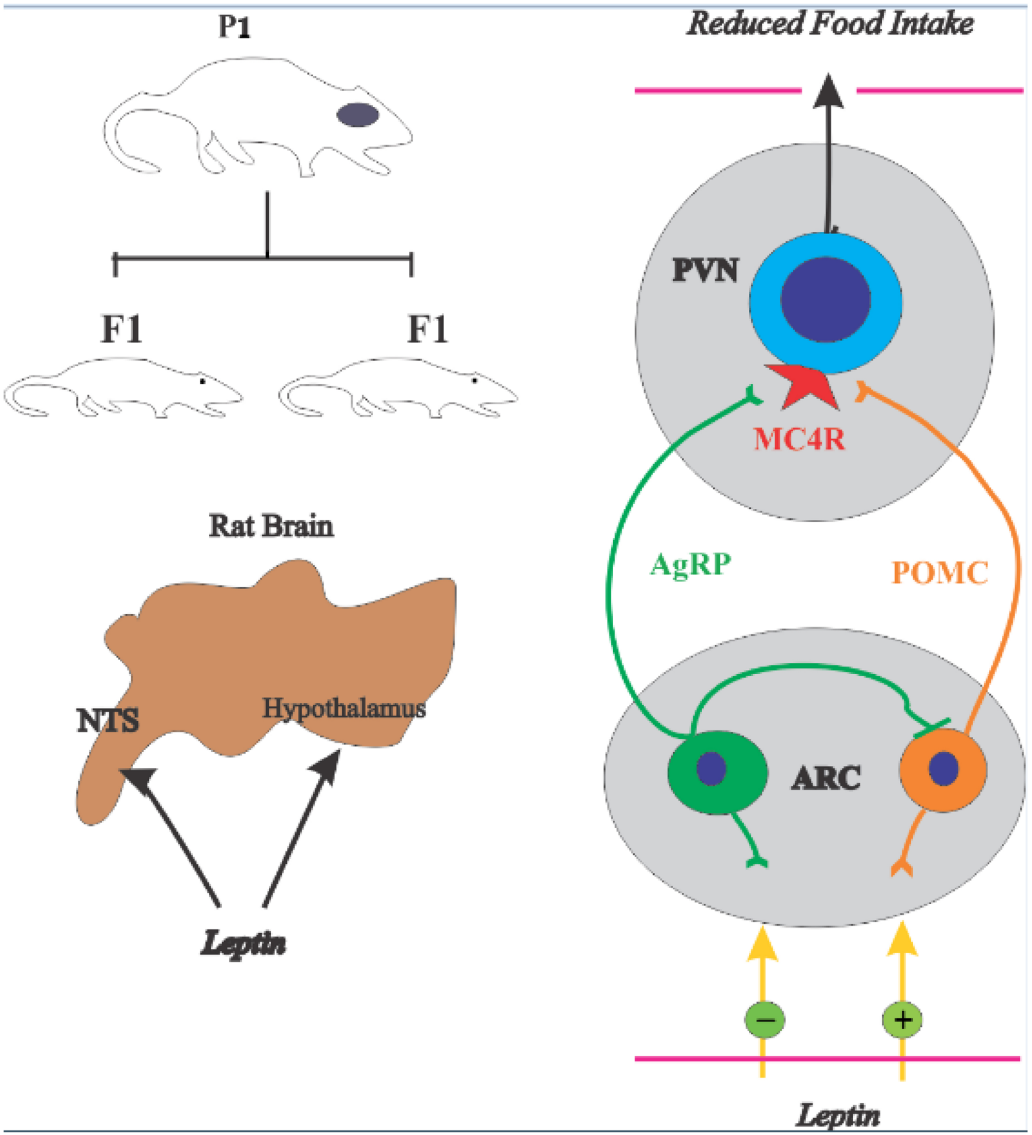
Control of food intake by a hypothalamic pathway The hypothalamus receives and incorporates neural, metabolic, and hormonal signals to regulate energy homeostasis. Adipocyte-derived hormone leptin and the melanocortin pathway have a critical role in the control of food intake **(A)**. AgRP, agouti-related protein; MC4R, melanocortin 4 receptor; POMC, pro-opiomelanocortin; PVN, paraventricular nucleus.

Hypothalamus contains two sets of first-order neurons which are considered the best leptin-responsive neurons in the brain [43]. The neurons coexpressing NPY and Agouti-related peptide (AgRP), and neurons coexpressing POMC and cocaine and amphetamine-related transcript (CART). The former stimulate food intake while the latter repress it. Both sets of neurons extend their projections in Arc to second-order neurons, melanocortin 4 receptor (MC4R) and other areas in the brain (Figure 7). NPY/AgRP neurons are negatively regulated by leptin (Figure 6), and fasting boosts the expression of NPY and AgRP [43, 44]. The study showed that deletion of AgRP neurons in mice triggered increased energy consumption and become mildly lean in late life [45]. In recent, NPY/AgRP neurons were ablated postnatally, using different genetic approaches, although the deletion of neurons in each study was contrasted noticeably [46]. The message was a clear loss of NPY/AgRP neurons in adult life led to severe fat deposition. NPY connection with leptin has been a presently debatable point in appetite research. NPY act through G protein-coupled receptor, named (Y1-Y5). Earlier data recognized that Y1 and Y5 are more effective receptors expressed in hypothalamus regulating NPY neurons in response to appetite signals [47]. It has been reported that Arc NPY expression is decreased by leptin injection [48]. Previous observations also showed that leptin hyperpolarized NPY neurons in the hypothalamus and block their neuronal signaling in Arc [49]. A study revealed that orexin, a hormone released by adipocytes effect Arc through neuronal projection from the lateral hypothalamic area, activates NPY neurons [50]. Therefore, our data indicate that HFD-F1 offspring had increased number of NPY positive neurons in the hypothalamus and increased leptin levels. However, the increased level of leptin is due to leptin resistance in HFD-F1 offspring. These results clarify that obese mother and lactation has an adverse effect on energy consumption and metabolic complication in next generation.

Earlier observations confirmed that energy homeostasis required STAT3 expression on NPY neurons in Arc [51]. A study has confirmed that deletion of STAT3 from NPY neurons in mice leads to hyperphagia, and specific depletion of STAT3 in NPY neurons in hypothalamus resulted in increased NPY mRNA level, which led to obese phenotype [52]. Activation of STAT3 involves phosphorylation of tyrosine residue (Tyr 705). A study revealed that phosphorylation of STAT3 at Tyr 705 is most commonly mediated by JAKs, especially Janus tyrosine kinase 2 (JAK2) [53]. Previous data demonstrated that JAK2 and STAT3 pathways trigger leptin action [54, 55]. We assumed that obesity in HFD-F1 due to leptin resistance. Diet insufficiency usually results from a decrease in plasma leptin concentration, and low plasma leptin levels activate a chain of neuroendocrine signals to upsurge food ingestion [56]. Previous results indicated that phosphoinositol 3 kinase activates downstream targets, which in turn coordinates the STAT3 to activate NPY gene expression [57]. These investigations showed that STAT3 and NPY might involve in leptin resistance in obese animals. In the present study, pSTAT3 in the Arc was identified by IF and IHC after leptin/saline administration in both Chow-F1 and HFD-F1 groups. Leptin treatment in Chow-F1 significantly decreased food intake and increased number of pSTAT3-IR cell bodies in the Arc, suggesting Chow-F1 had a normal leptin signaling. There were no significant changes in the food consumption and pSTAT3-IR in saline treated HFD-F1 offspring, indicating that HFD-F1 is more susceptible to leptin. Thus, central leptin signaling in HFD-F1 must be rigorously decreased because they exhibited low sensitivity although leptin levels are high. From the present study, we conclude that HFD feeding during pregnancy has adverse outcomes in their male offspring via hypothalamic leptin resistance.

Our work indicates that HFD feeding during pregnancy in female rats may alter hypothalamic feeding networks and transform energy metabolism of their offspring. In the present study, we concentrated on maternal obesity. We concluded that metabolic alterations during pregnancy affect hypothalamic neurogenesis of their male offspring and developed leptin resistance. In a count of gene level investigations of hypothalamus concerning externals cues, the connection between parents and offspring is required. An explanation of these biological relations would help us to understand better the exact mechanism of human brain functions and development of neuronal circuits. Inhibition of SCOS3, MC4R direct stimulation may recover leptin resistance and to investigate whether similar hypothalamic changes occur in the second filial generation or not needs further research.

## MATERIALS AND METHODS

### Experimental model

All animal treatments were performed in agreement with international ethical guidelines and the National Institutes of Health Guide for the Care and Use of Laboratory Animals. The experiments were directed with the approval of the Committee of Experimental Animal Administration School of Medicine, Zhejiang University. Six-week-old Sprague-Dawley (SD) rats weighing 140-160g were purchased from the Shanghai Experimental Animal Center (Chinese Academy of Sciences, Shanghai, China) and housed (12: 12 h dark/light cycle) in the Zhejiang University Animal Center, Hangzhou, China. After acclimatization for 2 weeks, female rats were randomly allocated into two groups: normal control diet (chow, n=20) or high-fat diet (HFD, n=20) (Supplementary Table 1). Virgin female SD rats were mated with normal male SD rats at 10 weeks and pregnant females were fed chow or HFD from 8 weeks to delivery. Pregnancy-onset was assessed by the presence of a copulation plug after overnight mating (designated as day 0 (D0) of pregnancy). Maternal blood lipid levels were measured on D20 of pregnancy. Pups from HFD mothers were fostered by control females until they were weaned. Plasma was separated immediately after blood collection and stored at −80°C for later analysis of leptin levels in serum and visceral fat (gonadal, retroperitoneal) and subcutaneous fat (inguinal) were collected under anesthesia (chloral hydrate 0.04g/kg, i.p.) after 12 hours of fasting and weighed.

### Hematoxylin and eosin staining of white adipose tissue (WAT)

White adipose tissues were post-fixed in 4% paraformaldehyde (PFA) for 48 h, ethyl alcohol dehydrated and paraffin embedded. The tissues were sectioned on a microtome (5μm) and then stained with hematoxylin and eosin. The sections were photographed and assessed under a light microscope (BX51; Olympus, Tokyo, Japan). The size of the adipocytes was quantified using image J, version 1.40 (NIH, Bethesda, MD, USA). In each rat, 80 random adipocytes from three sections were measured for cell size under 20x optical magnification and expressed as the mean adipocyte area.

### Hypothalamic mRNA extraction and qRT-PCR

Total RNA from the hypothalamic enriched area the Arc (bregma −1.22 to −2.54 mm) and NTS (bregma −6.36 to −7.76 mm) was extracted from SD male offspring (4 weeks old) using TRIzol reagent (Life Technologies Corporation), according to the manufacturer's recommendations. RNA was quantified on a NanoDrop ND-2000 (Thermo Electron). 3 μg of total RNA from each sample was reverse-transcribed to cDNA using random primers and a reverse transcription system (Takara Bio Inc., Otsu, Japan) in accordance with the manufacturer's instructions. After cDNA synthesis, quantitative real-time PCR was performed using iQTM SYBR Green Supermix (Bio-Rad, Hercules, CA, USA) in accordance with the manufacturer's instructions. The PCR conditions were one cycle of 95°C for 3 min, followed by 39 cycles of 95°C for 10 s and 60°C for 30 s. PCR reactions were carried out in a 25 μl of reaction buffer that included 12.5 μl of SYBR Green, 0.25 μl of forwarding primer, 0.25 μl of reverse primer, 2 μl of cDNA and 10 μl ddH_2_O and performed in triplicate for each sample in the Bio-Rad CFX96 Real-Time PCR system. The fluorescence intensity of each sample was measured at each temperature change to monitor amplification of the target gene. The quantity of target gene was normalized to the rat housekeeping gene glyceraldehyde-3-phosphate dehydrogenase (GAPDH). The primer sequences used for the PCR amplification were: GAPDH, 5′-GGATTTGGTCGTATTGGG-3′ and 5′-GGAAGATGGTGATGGGATT-3′; NPY, 5′-CCGC CACGATGCTAGGTAAC-3′-and 5′-CAGCCAGAATG CCCAAACAC-3′; POMC, 5′-AGGCGACGGAAGAGAA AAGA-3′ and 5′-AGATTGGAGGGACCCCTGT-3′; obRb, 5′-TTGTGTCCTACTGCTCGGAAC-3′ and 5′-TGTTTCAGGCTTTTGGAAATTCAGT-3′; and SOCS3 5′-GCGGGCACCTTTCTTATCC-3′, and 5′-TCCCCGAC TGGGTCTTGAC-3′.

### Immunofluorescence (IF)

Ten HFD-F1 and Chow-F1 rats (4 weeks) from each group were sedated with 10% chloral hydrate (0.5 ml/100 g i.p.) and injected transcardially with saline followed by borate-buffered 4% paraformaldehyde (4% PFA) (pH 7.4). The brains were removed and kept in 4% PFA for 48 h. Super frost/plus microscope slides (Fisher) slide fixed sections were washed for several hours in PBS and then deparaffinized with xylene. Thereafter, the sections were incubated in PBS containing 1% H_2_O_2_ for 10 min followed by 1-hour incubation in blocking solution (10% goat serum) and then incubated with the rabbit anti-NPY (Cat no. D7Y5A; Cell Signaling Technology, lnc; dilution 1:1000) antibody at 4-°C overnight. After washing, a biotinylated secondary antibody Rhodamine Red-X goat anti-rabbit (Cat. no 1702286, Molecular Probe, Eugene, Oregon) and ABC-elite solution (1:500 diluted in 0.1 M PBS, for 1 hour) were used. DAPI (4′,6-Diamidino-2-Phenylindole, Dihydrochloride, Cat. No. D1306, Thermo-Scientific, Inc, US) was used as the chromogen. Finally, Slides were air dried, cover slipped with DPX medium mountant. The sections were photographed and assessed under a Carl Zeiss-800 confocal microscope (Baden-Wurttemberg, Germany). Data are presented as an average number of NPY positive cells per section from three Arc and NTS sections in each animal. Control sections for the single immunofluorescent procedure included omission of the primary antibody from the immunostaining protocol, which resulted in a complete absence of staining for the corresponding antigen.

### Leptin sensitivity

To assess the differences in the anorexigenic effect of leptin between HFD-F1 and Chow-F1, the rats (4 weeks old) were fasted for 20.00 h on P29 to 08.00 h on day30, and then injected with saline or leptin (2 μg/g body weight, i.p.) at 08.00 h on P30 [23]. Immediately after the injection, the food was given, and the consumption of the food was measured at 1, 2, 6 and 12 h after injection. Some rats were killed at 1 h after injection (leptin/saline) for pSTAT3 IF. The detailed protocol for hypothalamic IF analysis is as described above. The rabbit anti-pSTAT3 antibody (Cat no. D155018; BBI Life Sciences, Sangon Biotech, Shanghai, China; dilution 1:500) was used in the present study. The pSTAT3 was visualized with Alexa flour 488 conjugated streptavidin (Cat no. 1812166, Molecular Probe, Eugene, Oregon) with DAPI as a chromogen. Slides were air dried and cover slipped with DPX medium mountant. The data are presented as the mean number of p-STAT3 positive cells per section from three Arc sections in each animal. Photomicrographs were taken by Carl Zeiss-800 confocal microscope and analyzed by image-j software (National Institutes of Health, Bethesda, MD, USA).

### Immunohistochemistry of hypothalamic NPY and pSTAT3

Chow-F1 and HFD-F1 offspring (n=8, each group) were killed by anesthesia and transcardially perfused with saline followed by 4% PFA. The brains were excised and placed in ice-cold 4% PFA for 4 h and then in 20% sucrose solution at 4C° overnight. For the detection of NPY immunoreactivity, 5 um of hypothalamic sections were processed for IHC. Sections were blocked in 20 % goat serum to reduced nonspecific binding. NPY was detected using rabbit anti-NPY (Cat no. D121051; BBI life sciences, Shanghai, China; dilution 1:1000). After washing, second antibody Anti-Neuropeptide Y IgG (Cat no. ab10980) was used. DAPI (4′,6-Diamidino-2-Phenylindole, Dihydrochloride, Cat. No. D1306, Thermo-Scientific, Inc, US) was used as the chromogen. For leptin resistance, Chow-F1 and HFD-F1 (n=6, 4 weeks), monitored for food intake and were challenged with leptin/saline administration (2 μg/g, i.p). Food intake was measured at 1, 2, 6 and 12 h after injection. The following day offspring were killed and whole hypothalami were removed for pSTAT3 immunohistochemical analysis. A detailed protocol is as described above. The rabbit anti-pSTAT3 antibody (Cat no. 9145; Cell Signaling Technology, Beverly, MA, USA; dilution 1:500) was used in the present study. Donkey Anti-Rabbit IgG secondary antibody (1:1000, Jackson Immuno Research Laboratories, West Grove, PA) against pSTAT3. Tissue was treated with Elite ABC Kit (Vector Labs, Burlingame, CA) for 1 h, and the signal developed by Histostain-plus DAB kit (Cat no. 85-9243; Invitrogen). The sections were photographed and assessed under a light microscope (BX51; Olympus).

### Western blots

Total protein from Arc and NTS were extracted by homogenization in ice-cold radioimmunoprecipitation assay buffer (phosphate-buffered saline, 1% NP40, 0.5% sodium deoxycholate, 0.1% sodium dodecyl sulfate, 1 mmol/l phenylmethylsulfonyl fluoride, aprotinin 5 μg/ml, leupeptin 10 μg/ml, pepstatin A 1 μg/ml and phosphatase inhibitors cocktail). Homogenates were centrifuged for 25 min at 14,000g, supernatants collected and extract normalized to total protein content. The protein concentration was measured by BCA protein assay reagent kit (Pierce, Rockford, IL, USA). Proteins were separated by 12% SDS-PAGE, transfer to nitrocellulose membrane (Bio-Rad, Hercules, CA, USA) and blots was blocked for 1 h in 5% milk. Blots were incubated with antibodies to NPY (ab91262), SCOCS3 (ab16030), POMC (ab94446), ObRb (ab5593) and pSTAT3 (ab5073) (Abcam, Cambridge, UK, 1:1000) overnight at 4C°, and then incubated with their respective secondary antibody for one hour. Expression was normalized to GAPDH (CW bio CW0266A, Beijing, China, 1:1000) and protein intensities were determined and analyzed using Odyssey® Imager (LI-COR, Lincoln, NE, USA).

### Blood hormones and biochemical assays

Blood samples were collected from the heart and allowed for 10 min at room temperature. After clotting centrifuge it for 5 min at 3000 g, and stored the serum at −80°C until use. Serum leptin levels were measured by using commercial ELISA kit (Catalog no. F1532, Shanghai Westang Technology, China). The lowest detectable level and intraassay variation of leptin were 0.25 ng/ml and 7.6%. Serum triglyceride, Glucose, total cholesterol, high-density lipoprotein and low-density lipoprotein were measured with a Biochemical Analyzer (Advia 2400 Chemistry System; Siemens, Munich, Germany).

### Statistical analysis

All values are expressed as the mean±SEM. Graphpad prism, version 5.0 (GraphPad Software Inc., San Diego, CA, USA) was used for the statistical analysis. The body weight, body length, food intake and white adipose weight were analyzed using an unpaired Student's t-test. In the study of leptin sensitivity, two-way ANOVAs were conducted for the factors of treatment (leptin, saline) and groups (HFD-F1, Chow-F1). *P* < 0.05 was considered statistically significant.

## SUPPLEMENTARY MATERIALS FIGURES AND TABLES


