# Supplementary material for: Hypertriglyceridemia in female rats during pregnancy induces obesity in male offspring via altering hypothalamic leptin signaling
Source: Oncotarget. 2017 Jun 16;8(32):53450–64. doi: 10.18632/oncotarget.18519 (PMC5581122; doi:10.18632/oncotarget.18519)
Supplement: Supplementary file 1 [file oncotarget-08-53450-s001.pdf]

Hypertriglyceridemia in female rats during pregnancy induces obesity in male offspring via altering hypothalamic leptin signaling

SUPPLEMENTARY FIGURES AND TABLE

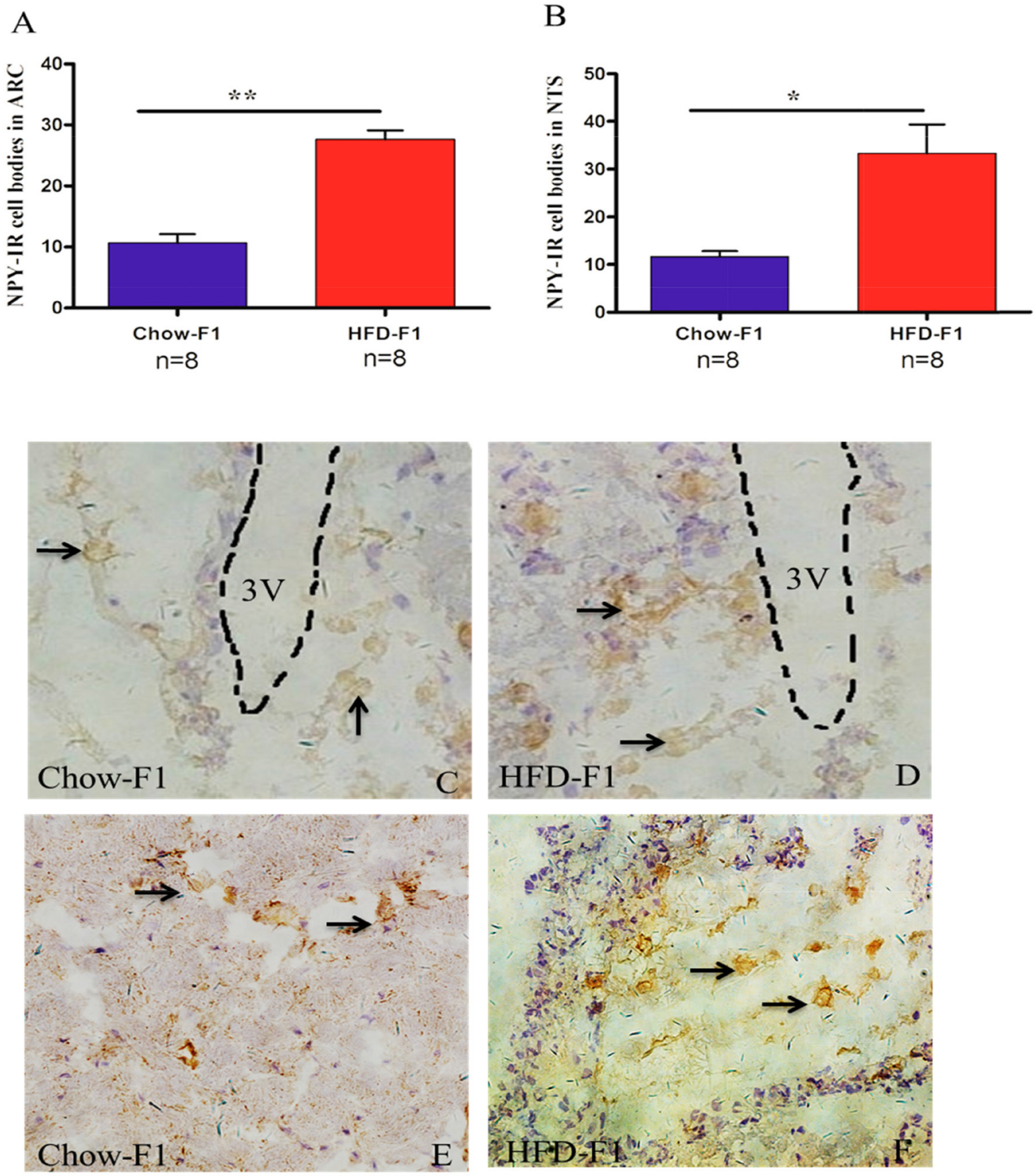

Supplementary Figure 1: NPY expression in the hypothalamus and brain stem. (A-B) Quantitative results of NPY positive neurons in the Arc and NTS. **(C-D)** NPY expression in the Arc of Chow-F1 and HFD-F1. **(E-F)** NPY expression in the NTS of Chow-F1 and HFD-F1. The data are expressed as the mean±SEM and the differences between the two groups were analyzed with Student's t-test. *p<0.05, **p<0.01.

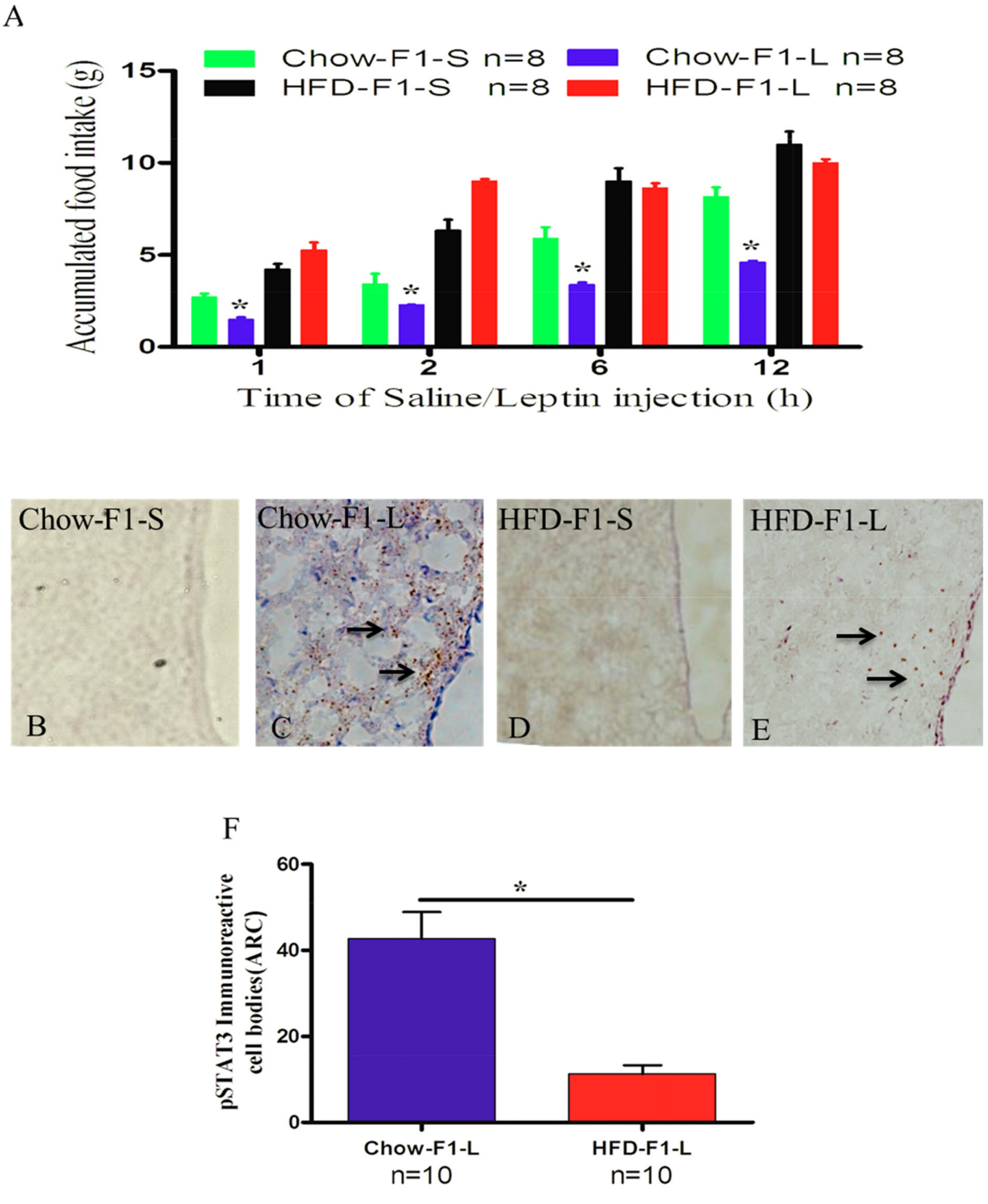

Supplementary Figure 2: HFD overfeeding during pregnancy in female rats induced leptin resistance in male offspring. Using a two-way ANOVA, food intake values at four time points were found to be significantly lower in Chow-F1 treated with leptin compared to all other groups. *$P < 0.05$. **(A)** Food intake 1, 2, 6 and 12 h after i.p. leptin (2 μg/g)/saline. **(B)** Expression of pSTAT3 in Chow-F1 after saline injection. **(C)** Expression of pSTAT3 in Chow-F1 after leptin injection. **(D)** Expression of pSTAT3 in HFD-F1 after saline injection. **(E)** Expression of pSTAT3 in HFD-F1 after leptin injection. **(F)** Quantitative results of pSTAT3 positive neurons after saline/leptin treatment. Data are expressed as the mean±SEM and the differences between the two groups were analyzed with Student's t-test. *$p<0.05$, **$p<0.01$.

Supplementary Table 1: Macronutrient compositions of a normal diet and a high-fat diet

Macronutrients	CD	HFD
Moisture (%)	9.2	6.9
Crude protein (%)	25.6	25
Crude fat (%)	4	32.4
Crude fiber (%)	3.8	2.9
Crude ash (%)	6.9	4
Carbohydrate (%)	50.5	28.8
FAs (g/100 g)	3.8	32.7
Saturated FAs (g/100g)	0.7	7.2
Monounsaturated FAs (g/100g)	0.95	21.7
Polyunsaturated FAs (g/100g)	2	3.8
Cholesterol (mg/100g)		12.9
Energy (kcal/100g)	340.4	506.8
Fat kcal (%)	10.6	57.5
Protein kcal (%)	30.1	19.7

CD, chow diet; HFD, high-fat diet; FAs, fatty acids.
